# Chemical Imaging of Pharmaceuticals in Biofilms for Wastewater Treatment Using Secondary Ion Mass Spectrometry

**DOI:** 10.1021/acs.est.2c05027

**Published:** 2023-05-02

**Authors:** Cecilia Burzio, Amir Saeid Mohammadi, Per Malmberg, Oskar Modin, Frank Persson, Britt-Marie Wilén

**Affiliations:** † Department of Architecture and Civil Engineering, Chalmers University of Technology, 41296 Gothenburg, Sweden; ‡ Department of Chemistry and Chemical Engineering, Chalmers University of Technology, 41296 Gothenburg, Sweden

**Keywords:** pharmaceutical, organic micropollutant, granular sludge, biofilm, wastewater, SIMS, mass spectrometry imaging

## Abstract

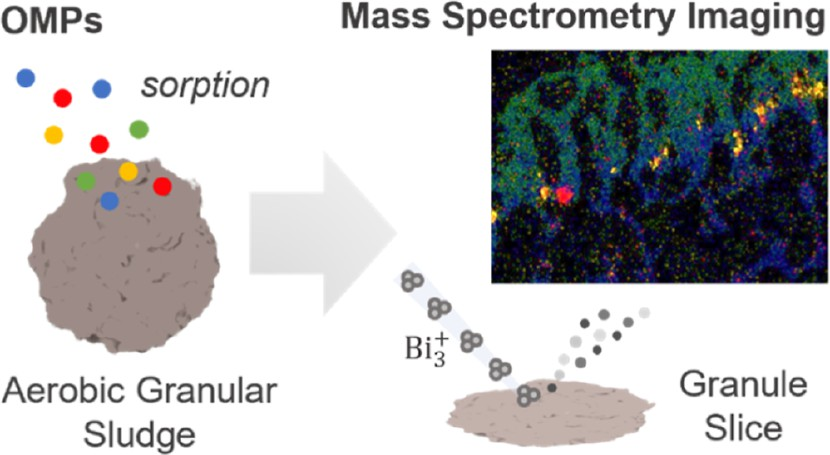

The occurrence of pharmaceuticals in the aquatic environment is a global water quality challenge for several reasons, such as deleterious effects on ecological and human health, antibiotic resistance development, and endocrine-disrupting effects on aquatic organisms. To optimize their removal from the water cycle, understanding the processes during biological wastewater treatment is crucial. Time-of-flight secondary ion mass spectrometry imaging was successfully applied to investigate and analyze the distribution of pharmaceuticals as well as endogenous molecules in the complex biological matrix of biofilms for wastewater treatment. Several compounds and their localization were identified in the biofilm section, including citalopram, ketoconazole, ketoconazole transformation products, and sertraline. The images revealed the pharmaceuticals gathered in distinct sites of the biofilm matrix. While citalopram penetrated the biofilm deeply, sertraline remained confined in its outer layer. Both pharmaceuticals seemed to mainly colocalize with phosphocholine lipids. Ketoconazole concentrated in small areas with high signal intensity. The approach outlined here presents a powerful strategy for visualizing the chemical composition of biofilms for wastewater treatment and demonstrates its promising utility for elucidating the mechanisms behind pharmaceutical and antimicrobial removal in biological wastewater treatment.

## Abstract 2

Removal mechanisms of organic micropollutants from wastewater are poorly understood. This study shows the localization and distribution of selected pharmaceutical residuals in the biological matrix of biofilms for wastewater treatment.

## Introduction

The occurrence in the aquatic environment of organic micropollutants (OMPs) consisting of pharmaceuticals, endocrine-disrupting compounds, pesticides, and personal care products is a global water quality challenge.^1^ OMP removal during conventional biological treatment of wastewater is usually incomplete, and discharge from wastewater treatment plants (WWTPs) is a major transport route of these compounds into nature.^2^ OMPs are usually detected at levels ranging from ng L^–1^ up to μg L^–1^ in domestic and hospital effluents,^3,4^ but concentrations of active pharmaceutical ingredients in wastewater from production facilities can reach up to the mg L^–1^ range.^5^

Physical, chemical, and biological mechanisms drive the removal of OMPs occurring in WWTPs, including biotransformation and sorption onto activated sludge and biofilms as dominant pathways of removal.^6^ Transformation driven by biological processes results in the modification of the chemical structure of the parent compound to metabolites or transformation products (TPs), which can be less biodegradable and more toxic than the parent compound.^7^ Sorption onto the sludge is a physicochemical process involved in binding ions or molecules from an aqueous solution onto the surface of a sorbent of biological origin. The interactions between the sludge surface and the micropollutant molecules govern the compound partitioning between the solid matrix and the water phase. Since these interactions occur at specific receptor sites distributed throughout the solid phase components and involve multiple sorbate functional groups, the characteristics of the biofilm matrix in the biological reactor [surface charge, specific surface area, extracellular polymeric substances (EPS), and mineral content] are also determining the degree of partitioning.^8,9^ As microorganisms and EPS, which make up the sludge and biofilms in biological wastewater treatment processes, are negatively charged at the surface,^10,11^ positively ionized micropollutants are likely to show the highest potential for sorption, due to electrostatic attraction, while hydrophobic compounds are expected to be attracted by the lipophilic cell membrane and cell wall of the microorganisms and the lipid fraction of the sludge.^12^

**Figure 1:**
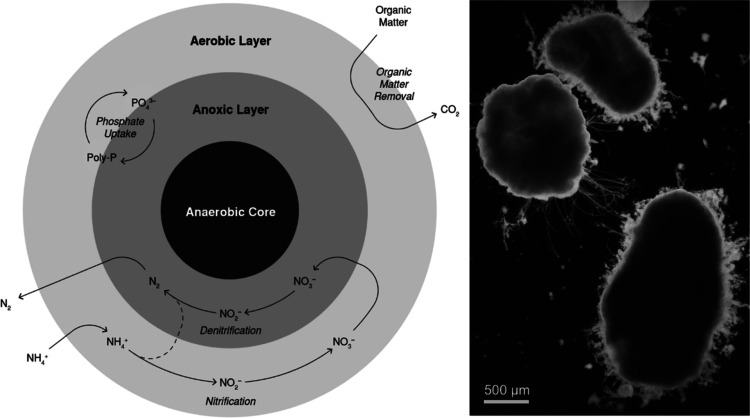
Schematic representation of an aerobic granule (left) with the simultaneous conversion processes for organic matter, nitrogen, and phosphorus, taking place within the different redox zones. A light microscopy picture (right) of the aerobic granular sludge used.

Among the biological technologies to treat wastewater at the WWTP, biofilm-based systems have been found to be more efficient compared to activated sludge in removing some pharmaceuticals such as diclofenac and trimethoprim.^13^ A more comprehensive understanding of the underlying mechanisms would facilitate the suitable choice of biological treatment techniques to achieve heightened removal efficiency. Granular sludge is an established biofilm technology, in which self-immobilized microorganisms embedded in a three-dimensional framework of EPS form compact, fast-settling, and dense aggregates of 200 μm up to a few millimeters in diameter (Figure 1). EPS are metabolic compounds, constituted mainly by polysaccharides, proteins, humic acids, uronic acids, nucleic acids, and lipids.^14,15^ EPS are responsible for the mechanical stability of biofilms, interconnecting and immobilizing bacterial cells. This biofilm structure incorporates water channels that allow for the transport of nutrients and electron acceptors. Within the granule, oxygen and substrate gradients are created, which are fundamental for the coexistence of a diverse population of microorganisms.^16^ Hence, nitrification, denitrification, biological phosphorus removal, and organic matter mineralization can occur simultaneously within the granule, in the different redox layers.^17^

The molecular pathways and the degradation mechanisms of OMPs are still not yet fully understood because of their biological and chemical complexity. For instance, information is lacking about where the transformation of OMPs occurs in engineered biological wastewater processes, i.e., bulk medium, inner biofilm layer, outer biofilm layer, or floc surface.^18^ Studies on *Pseudomonas aeruginosa* biofilms have revealed that some antimicrobial compounds are sequestered to the periphery and do not penetrate the biofilm, while others readily penetrate.^19^ EPS have also been proposed as key components in the partitioning and removal of OMPs, by offering numerous binding sites.^8^ However, the knowledge about the interaction mechanisms between EPS and OMPs is still very limited, and studies on this are needed to understand the fate of OMPs in biological wastewater treatment systems.^9^

To elucidate the molecular mechanisms behind the removal of OMPs, a key issue is to obtain the localization and spatial distribution of the parent compounds (or the TPs formed during the biotransformation) inside the biofilm matrix. Direct biochemical methods of imaging molecules in complex biological matrices are fundamental in the understanding of the biology of biofilms and the biochemical gradients within them.^20^ Time-of-flight secondary ion mass spectrometry (ToF-SIMS) is a highly sensitive surface analysis and label-free approach that can be applied to study the chemical composition of biological surfaces and thin films at high spatial resolution.^21^ A focused primary ion beam is sputtering secondary ions from the surface which are collected and analyzed by a mass spectrometer that measures their mass-to-charge ratio (*m*/*z*). In this way, the molecular composition (e.g., mass spectra) and lateral chemical distribution can be obtained at a depth of 1–2 nm. In the field of medicine and cell biology, SIMS has been used to analyze pharmaceutics, bacteria, bacterial biofilms, and the distribution of antimicrobials.^20,22−25^ However, it has never been used to examine the fate of OMPs in wastewater treatment systems or environmental biofilms. ToF-SIMS imaging was used as a label-free technique to explore and examine the localization of pharmaceuticals in the complex biological matrix of biofilm treating wastewater. The study aims to increase the understanding of the fate of OMPs in biofilm systems, with a focus on aerobic granules. Specific objectives were to (i) identify eight selected pharmaceuticals in the complex biological matrix of the biofilm; (ii) assess the distribution of the identified OMPs; and (iii) investigate molecular characteristics of endogenous components. The approach outlined here presents a powerful new strategy for chemical imaging of environmental biofilms for wastewater treatment and demonstrates its promising utility for elucidating the removal pathways of OMPs in biological processes.

## Materials and Methods

### Pharmaceutical Standard Analysis

A group of eight pharmaceuticals belonging to different therapeutic classes was selected for this study: the anticonvulsant carbamazepine; the fluoroquinolone antibiotic ciprofloxacin; two selective serotonin reuptake inhibitors (SSRIs) citalopram and sertraline; the selective serotonin and norepinephrine reuptake inhibitors venlafaxine; the non-steroidal anti-inflammatory diclofenac; the azole antifungal ketoconazole; and the angiotensin receptor antagonist losartan (Table S1). They are all common substances found in municipal wastewater effluents, surface waters, and dewatered sludge,^3,4^ exhibiting different biodegradability and sorption properties (Table S2).^4^ Pharmaceutical standard powders were individually mounted by sprinkling and pressing onto a 1 mm thick indium foil for ToF-SIMS analysis. All pharmaceutical standards were of analytical standard grade (Sigma-Aldrich), except for sertraline, which was purchased in pills from the pharmacy (Bluefish Pharmaceuticals).

### Batch Experiments

A lab-scale sequencing batch reactor (SBR) treating synthetic wastewater was used to cultivate the aerobic granular sludge. Details about the operations of the reactor can be found in the Supporting Information. The granules used for ToF-SIMS analysis were harvested from the SBR. One batch of granules was rinsed with deionized water, and stored at −80 °C, to serve as control biofilm without pharmaceutical exposure. Another batch of granules was exposed to a cocktail of the selected pharmaceuticals. Filtered (1.2 μm GF/C Whatman) effluent wastewater from the SBR was spiked with micropollutants without organic solvent (solvent evaporated before dosage) at the start of the experiment at a concentration of 10 mg L^–1^. This concentration was applied to overcome the limitation on ion yield and sensitivity of ToF-SIMS analysis and imaging for a biological sample with a complex matrix, as explained in detail later in the Results and Discussion section. The batch was aerated continuously, and the pH was controlled at 7.5 ± 0.3. After 6 hours of exposure to the OMP cocktail, the granules were harvested for SIMS analysis, rinsed with deionized water, and stored at −80 °C.

### Biofilm Sample Preparation

SIMS high-vacuum operating conditions (<10^–8^ mbar) require biological sample preparation. No fixation or embedding was used to preserve the native chemical composition and the spatial distribution of the targeted molecules.

To visualize the distribution of pharmaceuticals inside the biofilm, granule sections with 12 μm thickness were obtained using a microtome cryostat (Leica) at −20 °C. The cryosections were placed on indium titanium oxide (Sigma-Aldrich) coated glass and stored at −80 °C until analysis. To prevent any delocalization of the sample molecules,^21^ freeze drying of the granule slices was performed before ToF-SIMS analysis.

### ToF-SIMS Imaging and Data Analysis

The ToF-SIMS surface analysis was carried out using a ToF-SIMS 5 instrument (ION-ToF GmbH, Münster, Germany) equipped with Bi cluster ion gun as a primary ion source. Mass spectra in positive mode were acquired by using a 25 keV Bi_3_^+^ primary ion source. Charge compensation was carried out using low energy electrons.

Pharmaceutical references and biofilm slices were analyzed using the high current bunched mode with an achieved mass resolution of 6000 at *m*/*z* 500. Large area images were acquired using the stage scan function set at various ranges from 3500 μm × 4000 μm to 2000 μm × 2000 μm, at 800 by 800 pixels to 720 by 800 pixels using 6 frames/patch, 2 shots/frame/pixel. The pulsed primary ion currents were in the range of 0.25–0.30 pA. The spectra were internally calibrated to signals of common fragments as [C]^+^, [CH_2_]^+^, [CH_3_]^+^, and [C_5_H_15_NPO_4_]^+^ and high mass fragments as [C_16_H_13_Cl_2_]^+^, [C_17_H_18_NCl_2_]^+^, and [C_20_H_22_N_2_OF]^+^. The SURFACELAB software (version 7.2, ION-TOF) was used to process, record, analyze, and evaluate all the images and mass spectra.

### Scanning Electron Microscopy

After SIMS analysis, the dried biofilm structure and morphology were assessed by environmental scanning electron microscopy (SEM) using a Quanta 200 ESEM FEG (FEI Company, Oregon, USA) operated in high vacuum with a beam energy of 10 kV and a working distance of 12.6–13 mm. Biofilm sections were analyzed without surface modification to avoid generating artifact features on the biofilm surface.

### Peak Identification in the Biofilm Matrix

**Figure 2:**
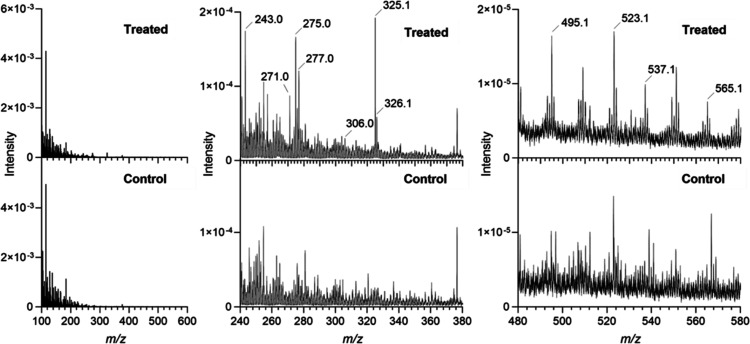
Comparison of the positive ion mode mass spectra for the treated (top) and control (bottom) biofilm. Treated and control mass spectra are illustrated in the range *m*/*z* 100–600 (left) and zoomed ranges of *m*/*z* 240–380 (middle) and 480–580 (right) are presented with the identified pharmaceutical peaks for better visualization. The intensities are normalized to the total ion count in the selected range of *m*/*z* 100–600.

To enable imaging of the distribution of pharmaceuticals in the complex biological matrix of aerobic granular sludge, the mass spectra obtained from control and treated biofilm samples were compared and analyzed (Figure 2). The ion peaks *m*/*z* that appeared in the treated but not in the control were identified with the assumption that those *m*/*z* peaks likely originated from the presence of pharmaceutical compounds. The identified *m*/*z* were then compared to the pharmaceutical standard spectra in which every pharmaceutical compound was represented by several molecular fragments in the mass spectrum (Figure S1). Assignments of the identified peak were performed mainly using the standard pharmaceutical mass spectra analyzed separately, with the deviation between the observed and theoretical peaks given as ppm (Table 1). Additionally, *m*/*z* peak screening of fragments and TPs was also performed by employing the defined mass spectra from previous studies and public repositories of mass spectra such as MassBank.^26^ To determine whether the assigned peaks originated from the actual pharmaceuticals or their metabolites after biotransformation, tandem mass spectrometry (MS/MS) analysis would be required. MS/MS capability is not common with SIMS instruments, but it is critical for accurately identifying the molecular nature and structural information of the ion peaks, which are beyond the scope of this study. The confidence in the identification of the pharmaceutical peaks in the biofilm matrix was based on (i) the pharmaceutical standard spectra, (ii) the literature values of peaks identified with MS/MS analysis, and (iii) the similar distribution in the treated biofilm of ion peaks representing the same pharmaceutical. To confirm that the identified peaks were generated by the presence of the pharmaceuticals and not by the natural biofilm variability among aerobic granules, an additional control biofilm was analyzed in a separate run (control 2). The detected peaks representing the identified pharmaceuticals were not observed in either of the controls. The spectra of the two control biofilms are presented in the Supporting Information, Figure S2.

**Table 1:** Assigned Peaks (*m*/*z*) for Pharmaceutical Identification Detected by SIMS in the Treated Biofilm

	assigned peaks					
compound	ion peak [*m*/*z*]	tentative ion species formula	ppm	detected in standards	references	MassBank accession number
citalopram	325.1	C_20_H_22_FN_2_O^+^	–48.6	X	(26, 27)	UFZ-UF411803
	326.1	C_19_[13]CH_22_FN_2_O^+^	14.7	X		Athens_Univ-AU151306
ketoconazole	495.1	C_26_H_28_ClN_4_O_4_^+^	–20.6	X	(28, 29)	
	523.1^a^	C_24_H_29_Cl_2_N_4_O_5_^+^	7.5			
	537.1^a^	C_24_H_27_Cl_2_N_4_O_6_^+^	32.6			
	565.1^a^	C_26_H_31_Cl_2_N_4_O_6_^+^	–60.5			
sertraline	275.0	C_16_H_13_Cl_2_^+^	–35.9	X	(26, 27, 30)	CASMI_2016-SM862703
	277.0	C_16_H_8_ClN_3_^+^	–48.7	X		Athens_Univ-AU150002
	306.0	C_17_H_18_Cl_2_N^+^	–92.4	X		

a Potential TPs.

## Results and Discussion

### Mass Spectrometry Measurements

For all the analyzed pharmaceutical standards, positive secondary ion mass spectra were obtained (Supporting Information Figure S1). Molecular ions and fragment ions were identified, and multiple mass spectrometry peaks were assigned to the pharmaceuticals for later detection in the biofilm matrix. [M + H]^+^, [M + Na]^+^, and [M + K]^+^ peaks representing molecular ions typically formed from the addition of hydrogen [H], sodium [Na], or potassium [K], commonly observed in the positive mode of ToF-SIMS spectra, were identified for all the compounds (Supporting Information Figure S1). Figure 2 presents the ToF-SIMS positive *m*/*z* spectra obtained for the control and treated biofilms. The positive ion spectra of the biofilm sections are dominated by peaks in the *m*/*z* 0–600 range. A number of pharmaceutical compounds could be identified within the treated biofilm matrix, namely, citalopram, ketoconazole, ketoconazole TPs, and sertraline. Peak assignments, based on pharmaceutical standard spectra (Figure S1), mass characteristics, and previous studies are provided in Table 1. All assigned ion peaks *m*/*z* could also be identified in the pharmaceutical standard spectra, with the exception of some ketoconazole TPs, that were identified from the literature.^28,29^ Carbamazepine, ciprofloxacin, diclofenac, losartan, and venlafaxine could not be identified in the biofilm using ToF-SIMS. The possible reasons are (i) low concentration of the pharmaceutical sorbed in the biofilm, (ii) biological transformation into unidentified TPs, (iii) weak signal intensity of the analyte, or (iv) high matrix effect suppressing the signal. The ionization efficiency of a compound can vary several orders of magnitude depending on the surrounding environment,^31^ therefore even if clear spectra were obtained from the pharmaceutical standards, it is possible that the presence of other molecules in the biofilm matrix hindered the ionization of the pharmaceutical compounds, and consequently their detection. There were also unidentified peaks, which were detected in the treated biofilm but not in the control (243.0, 271.0, 303.9, 465.1, 467.1, 509.1, 551.1, and 579.1).

### Distribution of Pharmaceuticals in the Biofilm Structure

**Figure 3:**
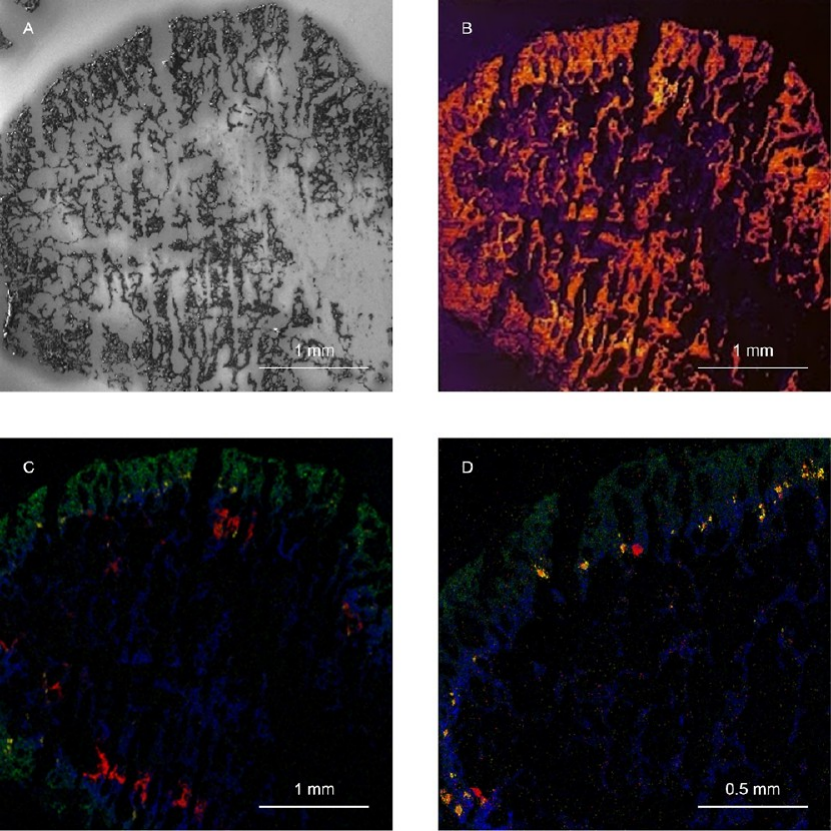
Images of the analyzed granule slice. SEM images of the treated biofilm section after SIMS analysis (A). Total ion image of the treated biofilm section (B). Overlay of ion distributions with different magnifications (C,D) of selected ion peaks *m*/*z* 275.0 in green (sertraline), 325.1 in blue (citalopram), 495.1 in yellow (ketoconazole), and 271.0 in red.

ToF-SIMS displays on the surface of the biofilm sections the intensity distribution of specific ions. The intensity of the selected ions can be illustrated in a two-dimensional map, resulting in ion images of the granule sections. Positive ion images with a 4 × 4 mm^2^ area were obtained from the treated biofilm (Figure 3). The pharmaceutical peaks detected with ToF-SIMS correlate with the biofilm matrix as indicated by the SEM images of the treated granule section (Figure 3A,C). The images revealed a porous structure in the biofilm (Figure S3). Channels are occurring naturally in the granules for water penetration, and they are observed in the SEM images. Hence, the granules contain an obvious amount of water, and freezing the specimen during the sample preparation can cause fracturing in the granule structure by forming ice and make it difficult to avoid cracks in the biofilm slices during the cryo-sectioning process. Embedding material for cryo-sectioning was not used to avoid any possible interference with the detected peaks in the mass spectrum.

**Figure 4:**
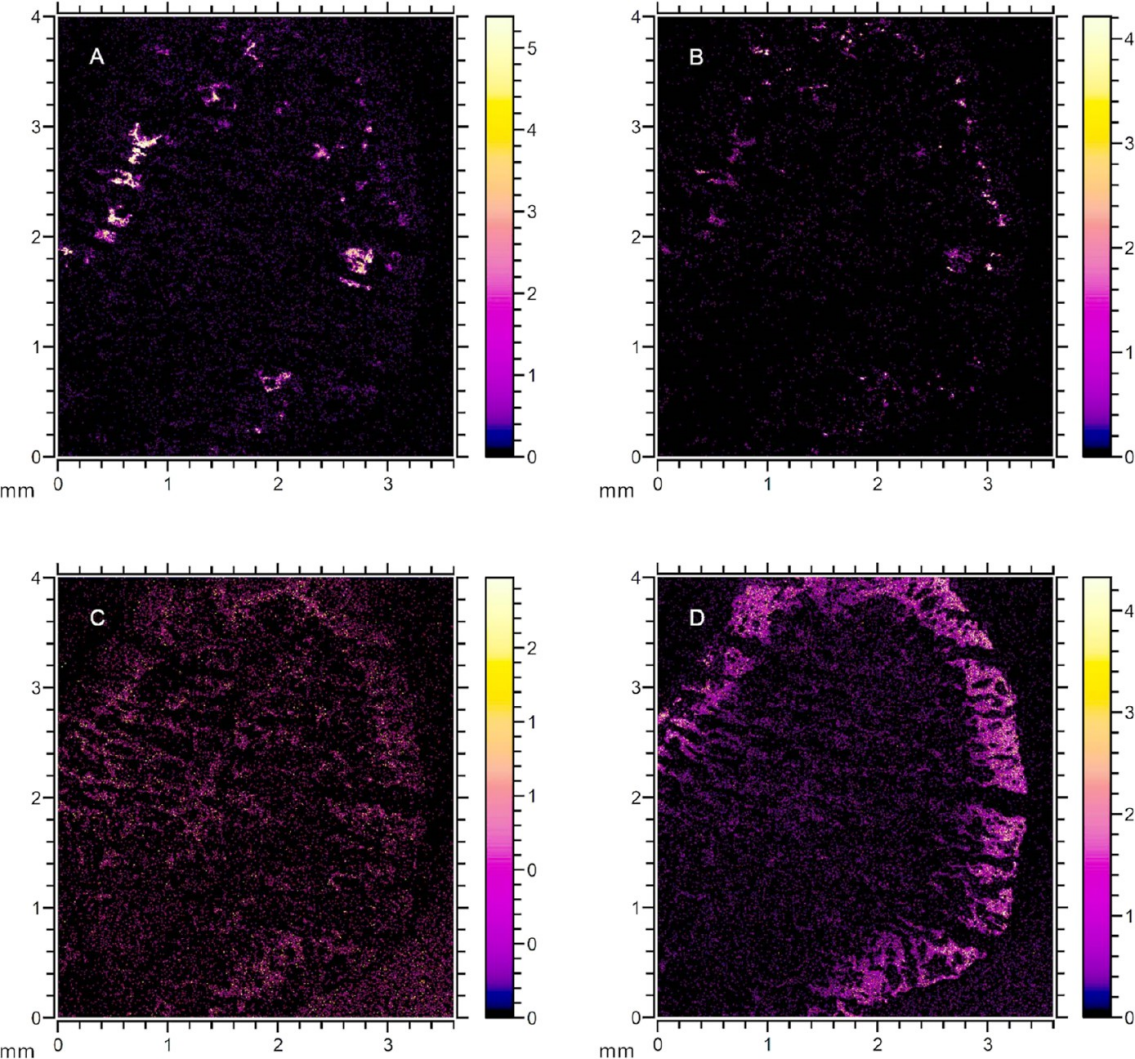
Images of representative *m*/*z* peaks obtained from the treated biofilm corresponding to (A) *m*/*z* 271.0, ketoconazole (B) at *m*/*z* 495.1, citalopram (C) at *m*/*z* 325.1, and sertraline (D) at *m*/*z* 275.0. The total ion counts for the selected *m*/*z* were 3.0 × 10^4^, 6.3 × 10^4^, 6.3 × 10^4^, and 7.8 × 10^3^, respectively. The color scale on the right indicates the relative SIMS signal intensity from high (white/yellow) to low (black/purple). To highlight the signal distribution, the images are scaled to their highest intensity. The images with the original color scale can be found in Supporting Information Figure S5.

Figure 4 presents the distribution of the four peaks throughout the treated biofilm section. The images revealed heterogeneity in the spatial distribution of different pharmaceuticals in the biofilm depth with local hot spots of higher intensity for some compounds. Peaks corresponding to the same pharmaceutical showed similar distributions (Supporting Information, Figure S4).

Citalopram was both detected at *m*/*z* 325.1 and 326.1 (Supporting Information Figure S4). The *m*/*z* 325.1 and 326.1 have previously been used as identification peaks for citalopram.^26,27^ Citalopram penetrated through the biofilm, with higher signal intensity observed in the depth of the granule (Figure 4C).

A peak corresponding to the parent compound [M – Cl]^+^ was observed at *m*/*z* 495.1. The SIMS images in Figure 4B show the distribution of this peak in several hotspots in the periphery of the biofilm. A similar distribution was observed at *m*/*z* 565.1 (Supporting Information Figure S4), corresponding to ketoconazole TP from the oxidative biotransformation at the dichlorophenyl imidazole moiety, which has also been observed in mouse and human liver microsomes and hepatocytes and mouse feces.^28,29^ Ion peaks *m*/*z* 523.1 and 537.1 (Supporting Information Figure S4) were also identified as potential ketoconazole TPs^28^ and matched the distribution of the parent compound. While ketoconazole biotransformation in wastewater has not been investigated yet, its metabolic pathways have been described for humans and rats.^28^ Other peaks were observed with similar distribution with the pattern of 14 Da differences, including *m*/*z* 509.1, 551.1, and 579.1 (Supporting Information Figure S6). The mass range of those peaks is well above the molecular weights of the pharmaceutical analyzed, suggesting the presence of other TPs.

Sertraline was detected at *m*/*z* 306.0 as molecular ion [M + H]^+^ and at 275.0 and 277.0 (Figures 4D and S4 Supporting Information). The *m*/*z* 275.0, 277.0, and 306.0 have been previously used as identification peaks for sertraline in previous studies.^26,27,30^ Based on the identified peaks, sertraline penetrated through the biofilm but was mainly bound in the periphery with higher intensity registered in the first 200 μm of the aerobic granule.

Previous research has identified peaks with *m*/*z* 243.0 and 271.0 (Figures 4A and S4) as fragments of ciprofloxacin and its metabolites,^26,32^ and the peaks were also observed in the ciprofloxacin standard spectra albeit with low intensity (Figure S1). To determine whether these fragments originate from ciprofloxacin or other organic compounds, further investigation using MS/MS would be required.

All the pharmaceuticals identified in the biofilm matrix are compounds commonly analyzed in the sewage matrix that accumulate in wastewater sludge at high concentrations, to a somewhat greater extent compared to the compounds not detected (Table S2). In biological processes at WWTP, sorption to sludge is considered the principal mechanism responsible for the removal of ciprofloxacin,^9^ ketoconazole,^33^ and sertraline.^30^

### Characterization of Biofilm Composition Using SIMS

**Figure 5:**
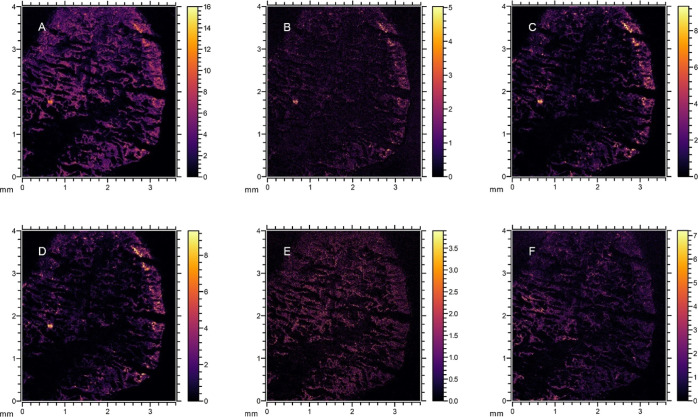
Ion images of the treated biofilm depicting localization of PC headgroup at *m*/*z* 86.1 (A), 166.0 (B), 184.0 (C), and 224.0 (D), adenine at *m*/*z* 136.0 (E), and lysine at *m*/*z* 147.1 (F). The total ion counts for *m*/*z* 86.1, 166.0, 184.0, 224.0, 136.0, and 147.1 were 8.4 × 10^5^, 1.6 × 10^5^, 2.5 × 10^5^, 6.9 × 10^4^, 2.2 × 10^5^, and 2.2 × 10^5^, respectively.

ToF-SIMS provided molecular recognition for endogenous biological molecules, enabling the visualization of constituents of EPS and cell components together with the pharmaceutical distribution in the biofilm (Figure 5). Assignments of the biofilm peaks, molecular formula, and references are described in Supporting Information Table S3. The ion images of the identified endogenous molecules for the control biofilms can be found in the Supporting Information, Figure S7.

The ion peaks *m*/*z* 86.0, 166.0, 184.0, and 224.0 (Figure 5) are the signature ions of the phosphocholine (PC) headgroup, a cell membrane phospholipid fragment.^34−36^ Lipids are commonly found in the EPS matrix of biofilm to treat wastewater,^15,37^ and they have been found to accumulate at the outer layer of aerobic granules.^38^ Lipids play an important role in the hydrophobic properties of sludge^39^ and they can help to emulsify the hydrocarbon substrate and increase its bioavailability.^15^

The ion peak *m*/*z* 136.0 (Figure 5E) was attributed to adenine, a nucleic acid marker for bacterial cytoplasm and extracellular DNA, which allows for imaging of bacterial distribution within the biofilm.^40^ The ion peak *m*/*z* 147.1 (Figure 5F) was identified as lysine, an amino acid. The adenine peak had higher intensity in the interior of the granule, possibly related to higher amounts of extracellular DNA. A previous study detected measurable levels of extracellular DNA in tightly bound EPS, not in loosely bound and soluble EPS which are presumably more associated with the outer granular layer.^41^

### Colocalization of Pharmaceuticals and Endogenous Compounds

The results revealed that the pharmaceuticals accumulated in different locations of the biofilm matrix, indicating that several mechanisms played a role in the sorption and transformation of the compounds in the granule structure.

Citalopram penetrated deeply into the structure of the biofilm, whereas sertraline was mainly sequestered in the outer layer of the granule. Ketoconazole and its TPs concentrated in small areas with relatively high intensity and did not penetrate the deeper layers of the biofilm. The biofilm matrix likely acts as a diffusion barrier, preventing antimicrobial substances from reaching the inner part of the granule.^42^ The EPS seem to play an important role in the sorption of antibiotics, thereby restricting the diffusion of compounds from the surrounding environment into the biofilm. Zhang et al.^8^ observed that protein-like substances such as tryptophan and tyrosine were involved in the sorption of ciprofloxacin and the binding was through carboxyl, amine, and hydroxyl groups in the EPS of sewage sludge. Moreover, because of its dense structure, the biofilm presents a gradient of redox conditions and different substrates, which are fundamental for the coexistence of a diverse population of microorganisms with different functions and characteristics (Figure 1). The outer layers of the granules are exposed to shear forces and high nutrient availability and therefore contain more fast-growing microorganisms than the inner core. Previous research has shown that activated sludge with a high growth rate (short solids retention time) is more negatively charged and less hydrophobic than sludge with a lower growth rate.^43^ The existence of various microenvironments and biofilm characteristics within the granule^37^ can influence the affinity and availability of sorption sites for pharmaceuticals.^9^ Therefore, the diverse interactions could also be attributed to the molecular structure and physical properties of the pharmaceuticals and the components of the biofilm found at different depths.

**Figure 6:**
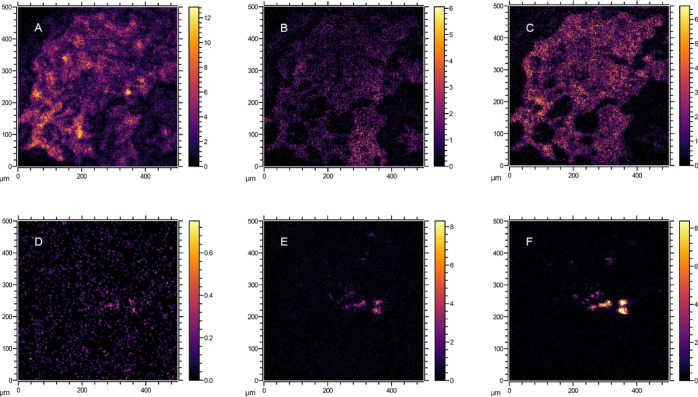
Ion images from the same scanned area of the biofilm showing the localization of the identified pharmaceuticals and endogenous molecules. On the upper row: PC headgroup (A) at *m*/*z* 184.0, citalopram (B) at *m*/*z* 325.1, and sertraline (C) at *m*/*z* 275.0. On the lower row: cholesterol (D) at *m*/*z* 369.3, *m*/*z* 271.0 (E), and ketoconazole (F) at *m*/*z* 495.1.

The buildup of ketoconazole in several hotspots with high intensity, rather than a more homogeneous distribution in the biofilm like for sertraline and citalopram, could indicate the accumulation in the vesicles of microorganisms. Amine-containing pharmaceuticals have also been shown to accumulate via ion trapping in the acidic vesicles, such as lysosomes and endosomes, of eukaryotic microorganisms and within the protozoic cells of the activated sludge community.^44^ Protozoa, such as ciliates, flagellates, and amoebae, are eukaryotes commonly present in wastewater sludge and they can make up over 9% of the volatile solids in activated sludge systems.^45^ Protozoa were also frequently observed in the cultivated granular sludge used as biofilm in this investigation (Supporting Information Figure S8). Since cholesterol is present in eukaryotic cell membranes but absent from prokaryotic cells,^44^ the distribution of the signature peak *m*/*z* 369.3 for cholesterol was visualized to show the colocalization of the pharmaceuticals and protozoan cell membranes (Figures 6 and S9 Supporting Information). The peak was found with low abundance at specific distributions consistent with the scale of single protozoa. Ketoconazole peaks likely colocalized with the cholesterol peak, suggesting that these compounds could accumulate in the lysosomes of the protozoa found embedded in the biofilm.

Sertraline and citalopram reflected similar distributions as the lipids and adenine, throughout the biofilm (Figures 4 and 5). Both these SSRIs have been observed to accumulate in several aquatic organism tissues, and mechanisms, other than hydrophobicity, have been suggested to contribute to the distribution of antidepressants in fish brains.^46^ Citalopram and sertraline are protonated at circumneutral pH; therefore, the sorption could be governed via electrostatic interactions with negatively charged biofilm sites.^47^ Not only do the cell walls of Gram-positive bacteria and the outer membrane in Gram-negative bacteria contain anionic lipid molecules, but also the plasma membranes are negatively charged due to the presence of anionic phospholipids.^48^ The colocalization of lipids and SSRIs could suggest that the sorption of citalopram and sertraline might occur through electrostatic attraction between the protonated amine and the anionic sites of the bacterial membrane and cell wall. Drug–lipid interactions are expected, as several pharmaceuticals have intracellular compartments as their site of action, and they need to cross the phospholipid bilayers to reach the intracellular targets.^49^ Antidepressants have been previously shown to accumulate in the lipid part of biological membranes and have high affinity binding to the lipid bilayers likely by Coulomb interactions.^50^ Citalopram and sertraline also showed complementary distribution in the biofilm slice (Figure 3). Higher intensity of citalopram was observed toward the core of the granule, whereas sertraline concentrated mainly in the periphery. This might suggest a competition for preferred binding sites.

The transport of molecules such as pharmaceuticals into heterogeneous biofilm structures is a complex process where the diffusion properties can differ due to voids, composition of the matrix, and variations in interactions between the pharmaceuticals and the biofilm constituents.^51^ The initial concentration of OMPs has been observed to affect the kinetics of biotransformation^52^ and the total sorbed pharmaceutical concentration depends on its concentration in the solution.^53^ The relatively high concentration of pharmaceuticals applied in this study (10 mg L^–1^) allowed us to observe mechanisms of removal, e.g., differences in the sorption capacity in the different parts of the granules. Similar interaction mechanisms should occur irrespective of the concentrations of pharmaceuticals applied.

### Challenges of SIMS Biological Analysis

SIMS imaging has been recognized as an effective technique for the spatial localization and chemical information of molecules in biological samples albeit with some technical limitations. In common SIMS techniques, extensive fragmentation of molecular species and low ionization yield are disadvantages due to a hard ionization method. SIMS is a desorption ionization method that applies a high-energy focused ion beam to produce secondary ions from the surface of the sample. Upon impact, the energy of the primary ion is transferred to the sample surface through a collision cascade. The energy of the primary ions is typically much higher than the bond energies of the molecules at the sample surface. This results in the large fragmentation of molecules, which makes it difficult to eject intact molecules from the sample surface. Most desorbed molecules are neutrals, and only around 1% of the ejected molecules are charged.^54−56^

SIMS is known to be a qualitative method rather than a quantitative analysis due to limitations of sensitivity and matrix effect in the complex biological samples including the granulated bacteria in this study.^56^ In such complex biological systems with differing compositions, the ion yield might be suppressed; therefore, the absence of an ion in a spectrum or ion image does not necessarily indicate the absence of the related molecule from a biological sample. On the other hand, the SIMS imaging technique with a high lateral resolution reduces the analysis pixel area and the amount of material available to ionize and measure. This is associated with sensitivity as an inherent limitation. Even with the attomole detection limit for common mass spectrometers, the measurable concentration of molecules would be in the milli-molar range that is restricting the detection to the most abundant biomolecules.^55,56^

It should be considered that the sample surface topography influences the mass resolution that can be obtained. Thus, to gain the best results within the analysis area, a relatively flat sample surface is required. Accordingly, the granule slices’ surfaces with a lack of an ultimate flat area due to the tearing artifacts could lead to a slight deviation in the observed mass resolution.^57^

### Implications

ToF-SIMS was successfully applied to identify and visualize pharmaceutical compounds in the complex biological matrix of biofilms for wastewater treatment. This approach offers a powerful strategy for chemical imaging of environmental biofilms and demonstrates its potential utility for understanding the removal pathways of antimicrobials and pharmaceuticals in the biological processes to treat wastewater. ToF-SIMS can be used to study mechanisms of sorption, transport, and biotransformation in the biofilm and highlight the interactions between endogenous molecules and pharmaceuticals. Understanding the role of these interactions is critical in developing and optimizing removal technologies. ToF-SIMS also provided molecular recognition for endogenous biological molecules, enabling the visualization of constituents of EPS and their distribution. This method could represent a strategy toward the characterization of biofilm and EPS composition properties for wastewater treatment. Application of high concentrations of pharmaceuticals (10 mg L^–1^) facilitates detection using ToF-SIMS and makes it possible to study removal mechanisms and differences in sorption capacity between microenvironments in biofilms. The lower thresholds for detection should be investigated in further studies.

## Supporting Information Available

Details on materials and methods, including reactor operation, wastewater composition, sludge inoculum; characteristics of selected pharmaceuticals; literature values of sorption coefficients; assigned peaks for endogenous molecule identification; mass spectra of pharmaceutical standards and control biofilms; SEM microscopy images and light microscopy images; and ion images of the treated biofilm section and the control biofilms (PDF)
