# Supplementary material for: Chemical Imaging of Pharmaceuticals in Biofilms for Wastewater Treatment Using Secondary Ion Mass Spectrometry
Source: Environ Sci Technol. 2023 May 2;57(19):7431–41. doi: 10.1021/acs.est.2c05027 (PMC10193524; doi:10.1021/acs.est.2c05027)
Supplement: Supplementary file 1 — es2c05027_si_001.pdf [file es2c05027_si_001.pdf]

Supporting information

Chemical imaging of pharmaceuticals in biofilms for wastewater treatment using Secondary Ion Mass Spectrometry

Cecilia Burzio [a*], Amir Saeid Mohammadi [a], Per Malmberg [b], Oskar Modin [a], Frank Persson [a], Britt-Marie Wilén [a]

[a] Department of Architecture and Civil Engineering, Chalmers University of Technology, 41296 Gothenburg, Sweden
[b] Department of Chemistry and Chemical Engineering, Chalmers University of Technology, 41296 Gothenburg, Sweden

* Corresponding author: [burzio@chalmers.se]

Number of Pages: 20

Number of Figures: 9

Number of Tables: 3

List of Supporting Information

Supporting Text

Reactor operation, wastewater composition, and sludge inoculum.

List of Tables

Table S1. Selected pharmaceuticals with their characteristics (mass, formula, pK_a, LogD, structure). The structures of the parent compound and the dominant form at pH 7.5 are both presented. Structure and compound characteristics are retrieved from Chemicalize (ChemAxon).

Table S2. Literature values of sorbed concentrations of pharmaceuticals in dewatered digested sludge, and solid-water partitioning coefficients (k_d) obtained with wastewater sludge solids. Standard deviations are marked (±).

Table S3. Assigned SIMS peaks (*m/z*) for biofilm characterization.

List of Figures

Figure S1. Positive secondary ion mass spectrum of pharmaceutical standard powders onto indium foil. In bold, the identified molecular peaks $[M+H]^+$, $[M+Na]^+$ and $[M+K]^+$.

Figure S2. Comparison of the ToF-SIMS mass spectra in the positive ion mode for the two control spectra 1 (in blue) and 2 (in red). The full mass spectra are presented in the range *m/z* 100-600 (top) and for better visualization zoomed ranges of *m/z* 240–380 (middle) and 480-580 (bottom) are illustrated with the identified pharmaceutical peaks.

Figure S3. SEM microscopy images of the treated biofilm section. Images are from the biofilm analyzed with SIMS and are shown at different magnifications.

Figure S4. Distribution of identified peaks in the treated biofilm. Ion images of peaks *m/z* 243.0 (A) and 271.0 (B) with total ion counts of $5.1 \cdot 10^4$ and $3.0 \cdot 10^4$ respectively. Ion images of citalopram at peaks *m/z* 325.1 (C) and 326.1 (D) with total ion counts of $6.3 \cdot 10^4$ and $2.4 \cdot 10^4$ respectively. Ion images of sertraline at peaks *m/z* 275.0 (E), 277.0 (F), and 306.0 (G) with total ion counts of $6.4 \cdot 10^4$, $5.6 \cdot 10^4$, and $2.0 \cdot 10^4$ respectively. Ion images of ketoconazole at peak *m/z* 495.1 (H), and its transformation products at peaks 565.1 (I), 523.1 (J), and 537.1 (K) with total ion counts of $7.9 \cdot 10^3$, $3.5 \cdot 10^3$, $8.2 \cdot 10^3$, and $4.8 \cdot 10^3$ respectively. The color scale on the right indicates the relative SIMS signal intensity from high (white/yellow) to low (black/purple).

Figure S5. Images of representative *m/z* peaks obtained from the treated biofilm corresponding to *m/z* 271.0 (A), ketoconazole (B) at *m/z* 495.1, citalopram (C) at *m/z* 325.1, and sertraline (D) at *m/z* 275.0. The total ion counts for the selected *m/z* were $3.0 \cdot 10^4$, $6.3 \cdot 10^4$, $6.3 \cdot 10^4$, and $7.8 \cdot 10^3$, respectively. The color scale on the right indicates the relative SIMS signal intensity from high (white/yellow) to low (black/purple).

Figure S6. Distribution of unidentified peaks *m/z* 509.1 (A), 551.1 (B), and 579.1 (C) in the treated biofilm. Their distribution matches the peaks corresponding to ketoconazole and its TPs, suggesting that those might be fragments of ketoconazole metabolites. The color scale on the right indicates the relative SIMS signal intensity from high (white/yellow) to low (black/purple). The total ion counts for the selected *m/z* were $4.9 \cdot 10^3$, $4.5 \cdot 10^3$, and $1.3 \cdot 10^3$, respectively.

Figure S7. Ion images of the two control biofilms (1 and 2) depicting the total ion image (A), the distribution of adenine at *m/z* 136.0 (B), and lysine at *m/z* 147.1 (C), and the localization of phosphocholine

headgroup at *m/z* 86.1 (D), 166.0 (E), 184.0 (F) and 224.0 (G). The total ion counts for *m/z* 136.0, 147.1, 86.1, 166.0, 184.0, and 224.0 in control 1, were $6.2 \cdot 10^4$, $1.3 \cdot 10^5$, $5.7 \cdot 10^5$, $9.9 \cdot 10^4$, $1.9 \cdot 10^5$, and $3.2 \cdot 10^4$, respectively. The total ion counts for *m/z* 136.0, 147.1, 86.1, 166.0, 184.0, and 224.0 in control 1, were $2.0 \cdot 10^5$, $2.1 \cdot 10^5$, $2.0 \cdot 10^6$, $1.5 \cdot 10^5$, $3.5 \cdot 10^5$, and $6.4 \cdot 10^4$, respectively.

Figure S8. Light microscopy images of the cultivated aerobic granules with visible protozoa on the surface and within the matrix of the biofilm. The image on the left (A) was taken at 2x magnification (scale bar 500 µm). The image on the right (B) was taken at 20x magnification (scale bar 50 µm).

Figure S9. Ion images of peaks *m/z* 369.3 corresponding to cholesterol (A), *m/z* 271.0 (B), and *m/z* 565.1 representing ketoconazole (C). Overlay of the three peaks *m/z* 369.3, 271.0, and 565.1 (D).

Reactor operation, wastewater composition, and sludge inoculum.

A mineral composition with multiple organic carbon sources was used with a slight modification of the recipe used by Layer et al. [1]. Acetate, propionate, glucose, and peptone from enzymatic digest provided the same amounts of COD equivalents. The synthetic wastewater consisted of 128 mg L^{-1} $NaCH_3COO$, 80 mg L^{-1} CH_3CH_2COONa, 94 mg L^{-1} $C_6H_{12}O_6$, 81 mg L^{-1} peptone from enzymatic digest, 144 mg L^{-1} NH_4Cl, 16 mg L^{-1} $MgSO_4 \cdot 7H_2O$, 18 mg L^{-1} $CaCl_2$, 33 mg L^{-1} KCl, 13 mg L^{-1} KH_2PO_4, 17 mg L^{-1} K_2HPO_4, 200 mg L^{-1} $NaHCO_3$. Micronutrient solution contained 0.05 g L^{-1} H_3BO_3, 0.05 g L^{-1} $ZnCl_2$, 0.03 g L^{-1} $CuCl_2$, 0.05 g L^{-1} $MnSO_4 \cdot H_2O$, 0.05 g L^{-1} $(NH_4)_6Mo_7O_{24} \cdot 4H_2O$, 0.05 g L^{-1} $AlCl_3$, 0.05 g L^{-1} $CoCl_2 \cdot 6H_2O$, and 0.05 g L^{-1} $NiCl_2$ and 1 mL L^{-1} wastewater was added. The reactor was inoculated with granular sludge from a full-scale WWTP located in Strömstad (Sweden), which performs biological carbon, nitrogen, and phosphorus removal.

Table S1. Selected pharmaceuticals with their characteristics (mass, formula, pK_a, LogD, structure). The structures of the parent compound and the dominant form at pH 7.5 are both presented. Structure and compound characteristics are retrieved from Chemicalize (ChemAxon).

Pharmaceutical	Structure of the parent compound	Theoretical mass [Da]	Formula	pK_a	LogD at pH 7.5	Dominant form at pH 7.5
**Carbamazepine**	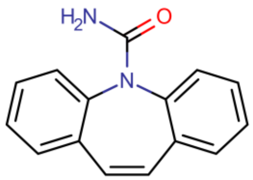	236.09	$C_{15}H_{12}N_2O$	15.96	2.77	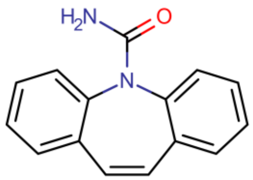
**Ciprofloxacin**	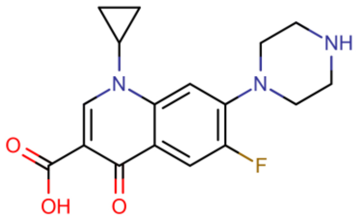	331.13	$C_{17}H_{18}FN_3O_3$	5.33, 8.77	-0.87	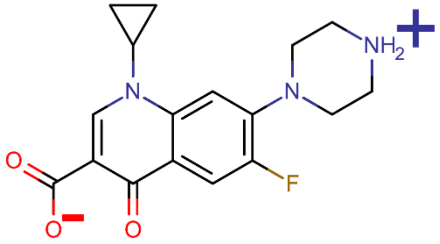
**Citalopram Hydrobromide**	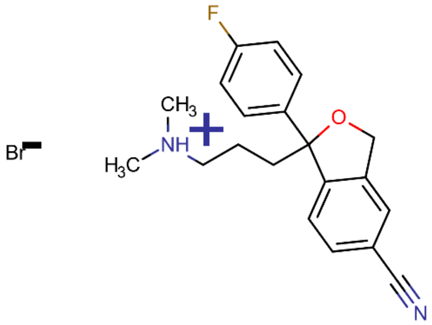	404.08	$C_{20}H_{22}BrFN_2O$	9.78	1.50	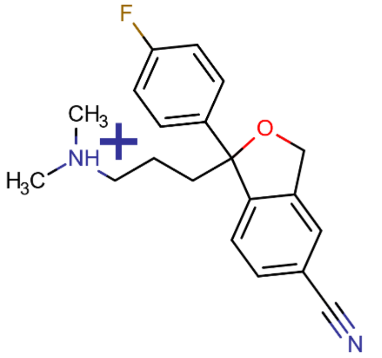

**Diclofenac Sodium**	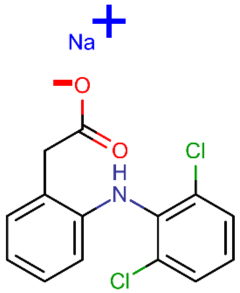	318.13	$C_{14}H_{10}Cl_2NNaO_2$	4, -0.58	1.04	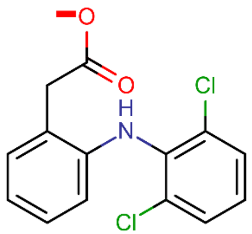
**Ketoconazole**	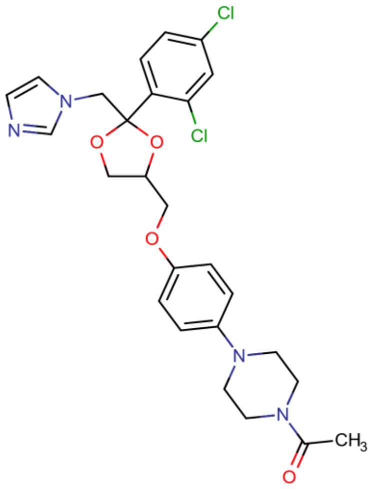	530.14	$C_{26}H_{28}Cl_2N_4O_4$	3.96, 6.42	4.16	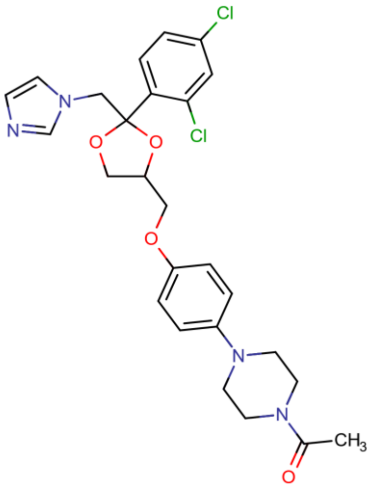
**Losartan Potassium**	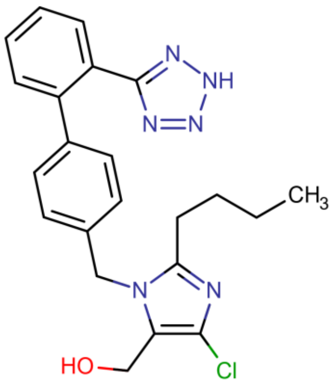	461.0	$C_{22}H_{22}ClKN_6O$	5.85, 3.85	3.77	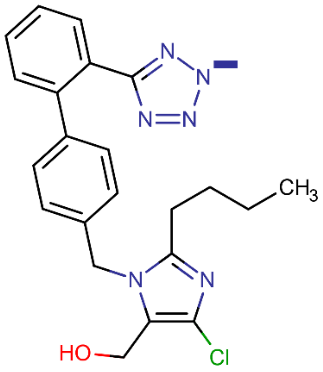

**Sertraline**	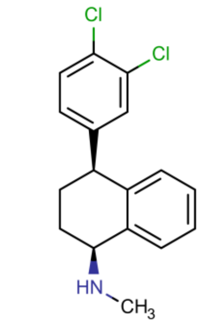	305.07	$C_{17}H_{17}Cl_2N$	9.56	3.11	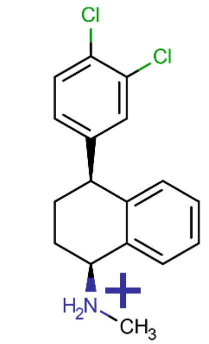
**Venlafaxine**	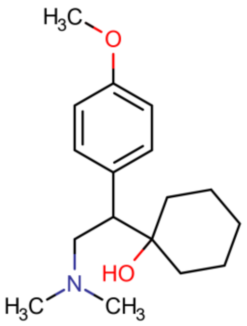	277.20	$C_{17}H_{27}NO_2$	9.06, 14.42	1.17	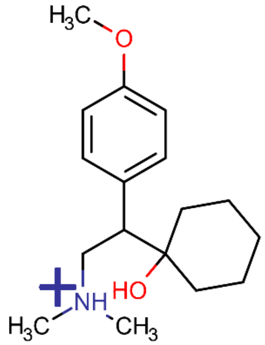

Table S2. Literature values of sorbed concentrations of pharmaceuticals in dewatered digested sludge, and solid-water partitioning coefficients (k_d) obtained with wastewater sludge solids. Standard deviations are marked (±).

	**Concentration in sludge**	k_d	**Median k_d**
**Compound**	**[µg kg^{-1}]**	**[L kgSS^{-1}]**	**[L kgSS^{-1}]**
Carbamazepine	89-190 [11]	1.7-1.8 [2], 36-50 [3], 2.7 [4], 1.2±0.5 [5], 17±1 [6], 135±39 [7], <2.7 [8]	2.7
Ciprofloxacin	68-450 [11], 4625[12]	4.2 [2], 12.25·10^3 [9]	6127.1
Citalopram	93-640 [4*], 460-760 [11]	2.1 · 10^2 [10], 3.2 [4]	106.6
Diclofenac	46-260 [4*], <LOQ-59 [11]	3.1-3.2 [2], <30 [3], 16±3 [5], 118±95 [7], 32-78 [8]	30
Ketoconazole	510-1800 [11], 2835 [12]	8.5 · 10^3 [10],	8500
Losartan	58-373 [4*]	2.4 [4]	2.4
Sertraline	170-910 [4*], 380-770 [11]	1.7 · 10^4 [10], 4.4 [4]	8502.2
Venlafaxine	20-203 [4*], 86-310 [11]	1.0 · 10^2 [10]	100

*dewatered sludge

Table S3. Assigned SIMS peaks (*m/z*) for biofilm characterization.

**Compound**	**Ion peak identified in biofilm [*m/z*]**	**Ion peaks identified in literature [*m/z*]**	**Formula**
Phosphocholine headgroup	86.1	86.1 [13], 86 [14]	$C_5H_{12}N^+$
Adenine	136.0	136.0 [15], 136.1 [14]	$C_5H_6N_5^+$
Lysine	147.1	147.11 [15]	$C_6H_{15}N_2O_2^+$
Phosphocholine headgroup	166.0	166 [14]	$C_5H_{13}NPO_3^+$
Phosphocholine headgroup	184.0	184.0 [13],184.1 [14], 184.08 [16]	$C_5H_{15}NPO_4^+$
Phosphocholine headgroup	224.0	224.1 [14]	$C_8H_{19}NPO_4^+$
Cholesterol	369.3	369 [13], 369.3 [14]	$C_{27}H_{45}^+$

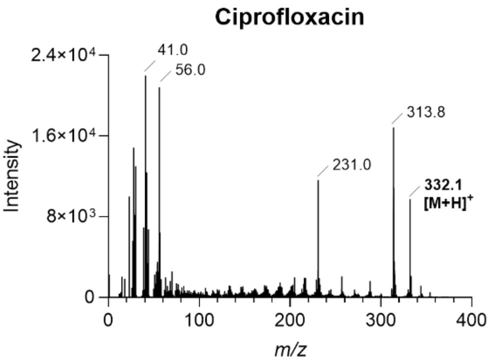
Ciprofloxacin
Intensity
m/z
41.0
56.0
313.8
231.0
332.1 [M+H]+

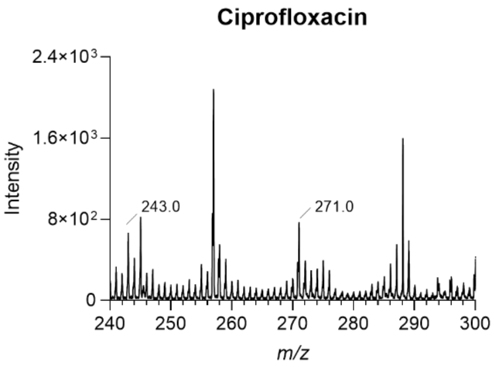
Ciprofloxacin
Intensity
m/z
243.0
271.0

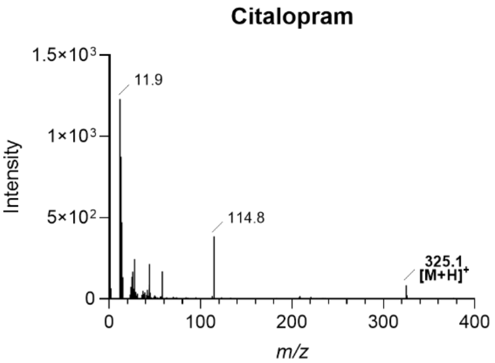
Citalopram
Intensity
m/z
11.9
114.8
325.1 [M+H]+

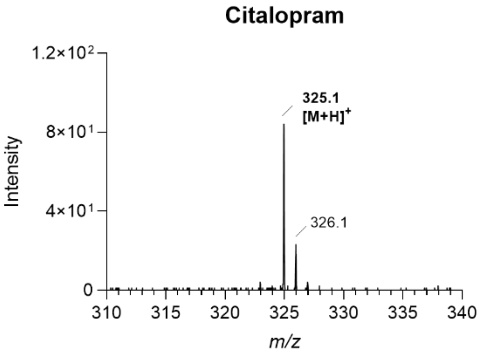
Citalopram
Intensity
m/z
325.1 [M+H]+
326.1

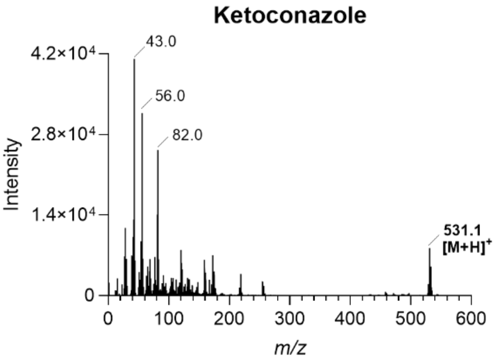
Ketoconazole
Intensity
m/z
43.0
56.0
82.0
531.1 [M+H]+

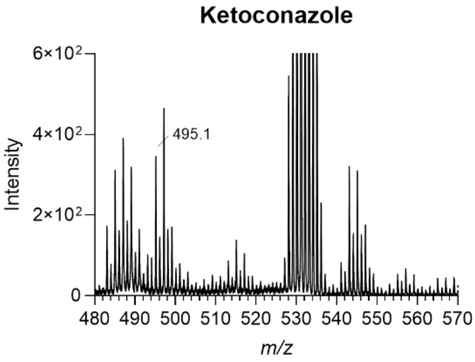
Ketoconazole
Intensity
m/z
495.1

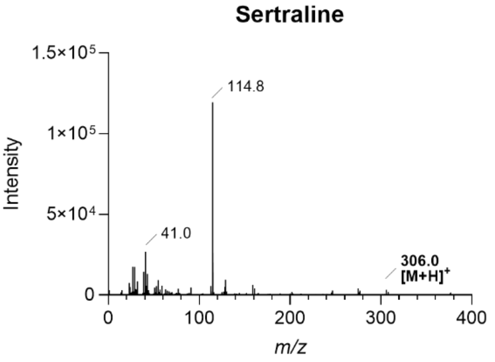
Sertraline
Intensity
m/z
114.8
41.0
306.0 [M+H]+

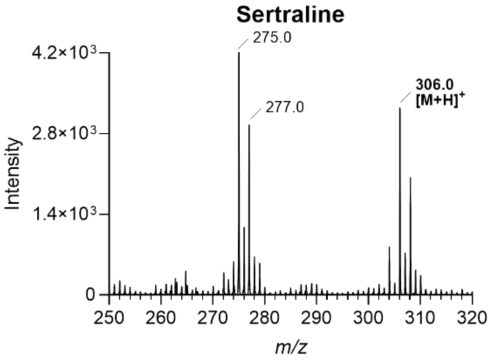
Sertraline
Intensity
m/z
275.0
277.0
306.0 [M+H]+

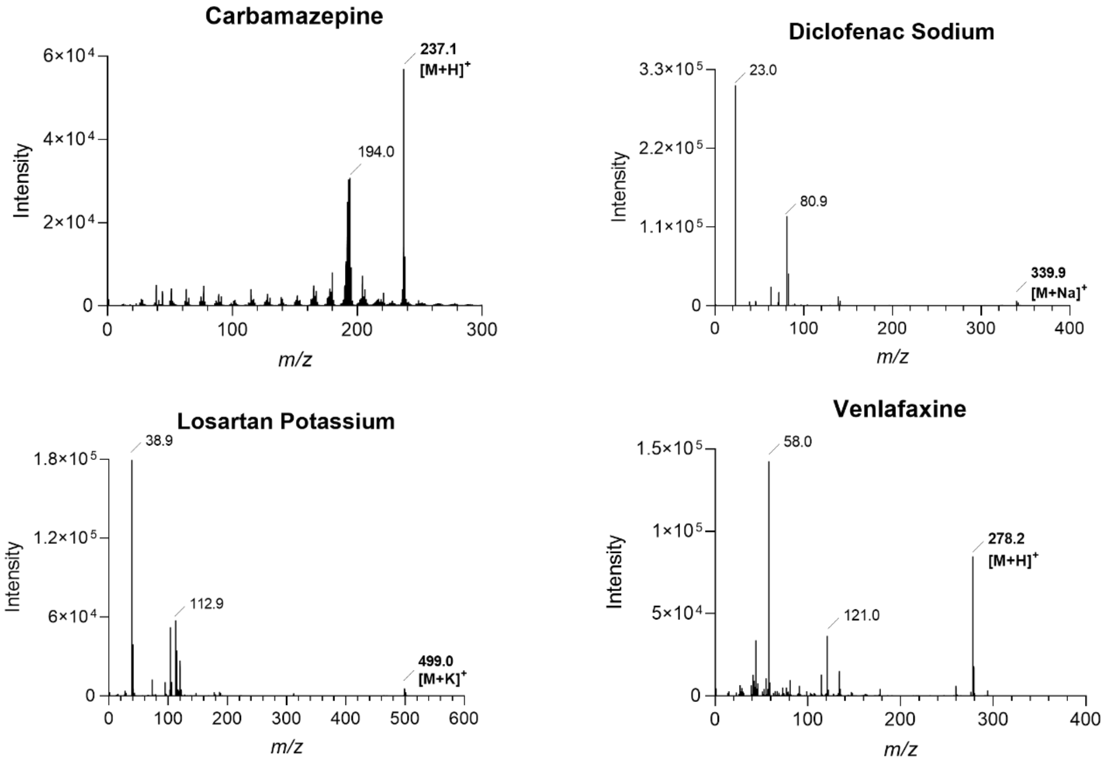

Figure S1. Positive secondary ion mass spectrum of pharmaceutical standard powders onto indium foil. In bold, the identified molecular peaks $[M+H]^+$, $[M+Na]^+$ and $[M+K]^+$.

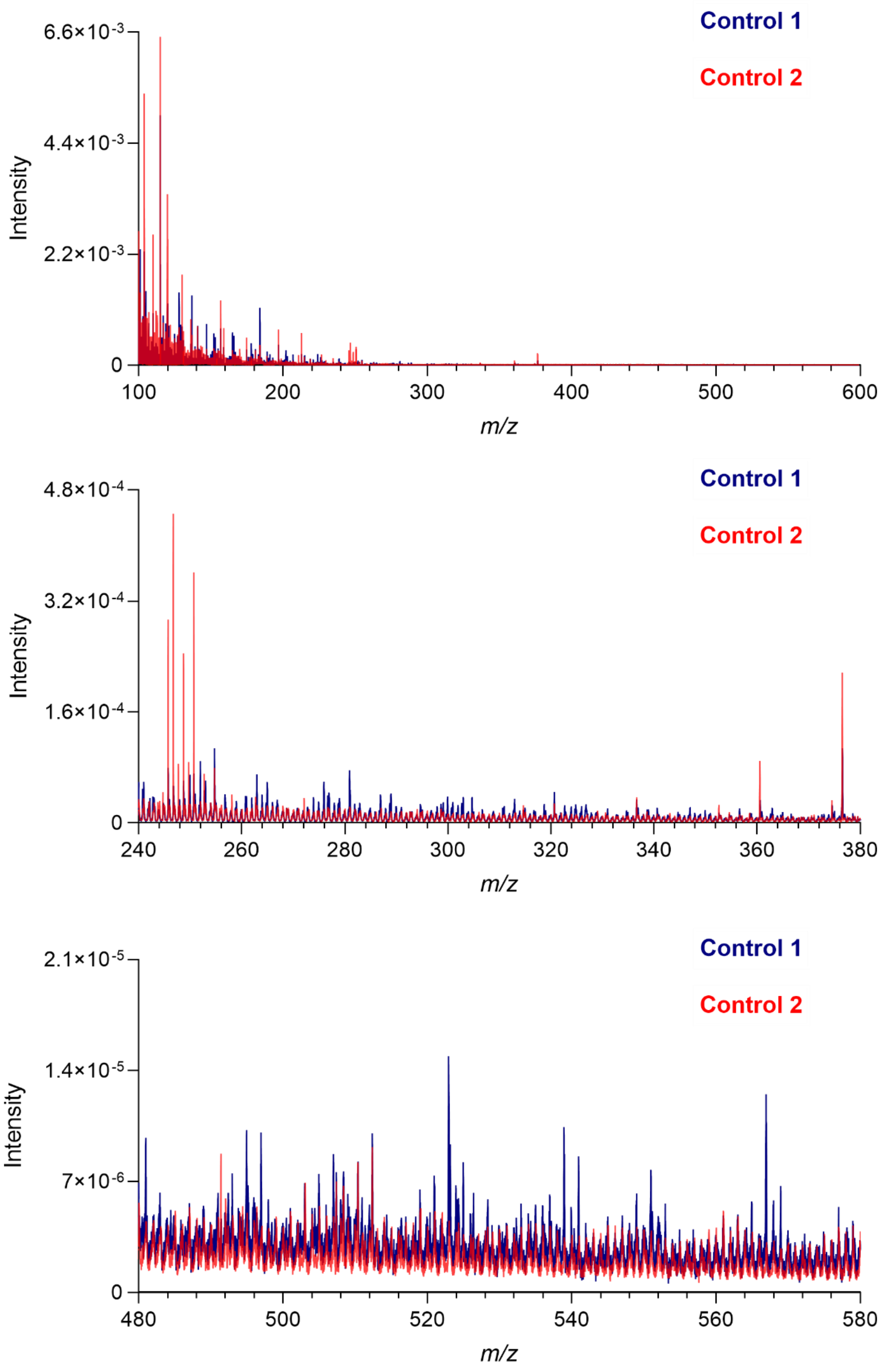

Figure S2. Comparison of the ToF-SIMS mass spectra in the positive ion mode for the two control spectra 1 (in blue) and 2 (in red). The full mass spectra are presented in the range *m/z* 100-600 (top) and for better visualization zoomed ranges of *m/z* 240–380 (middle) and 480-580 (bottom) are illustrated with the identified pharmaceutical peaks.

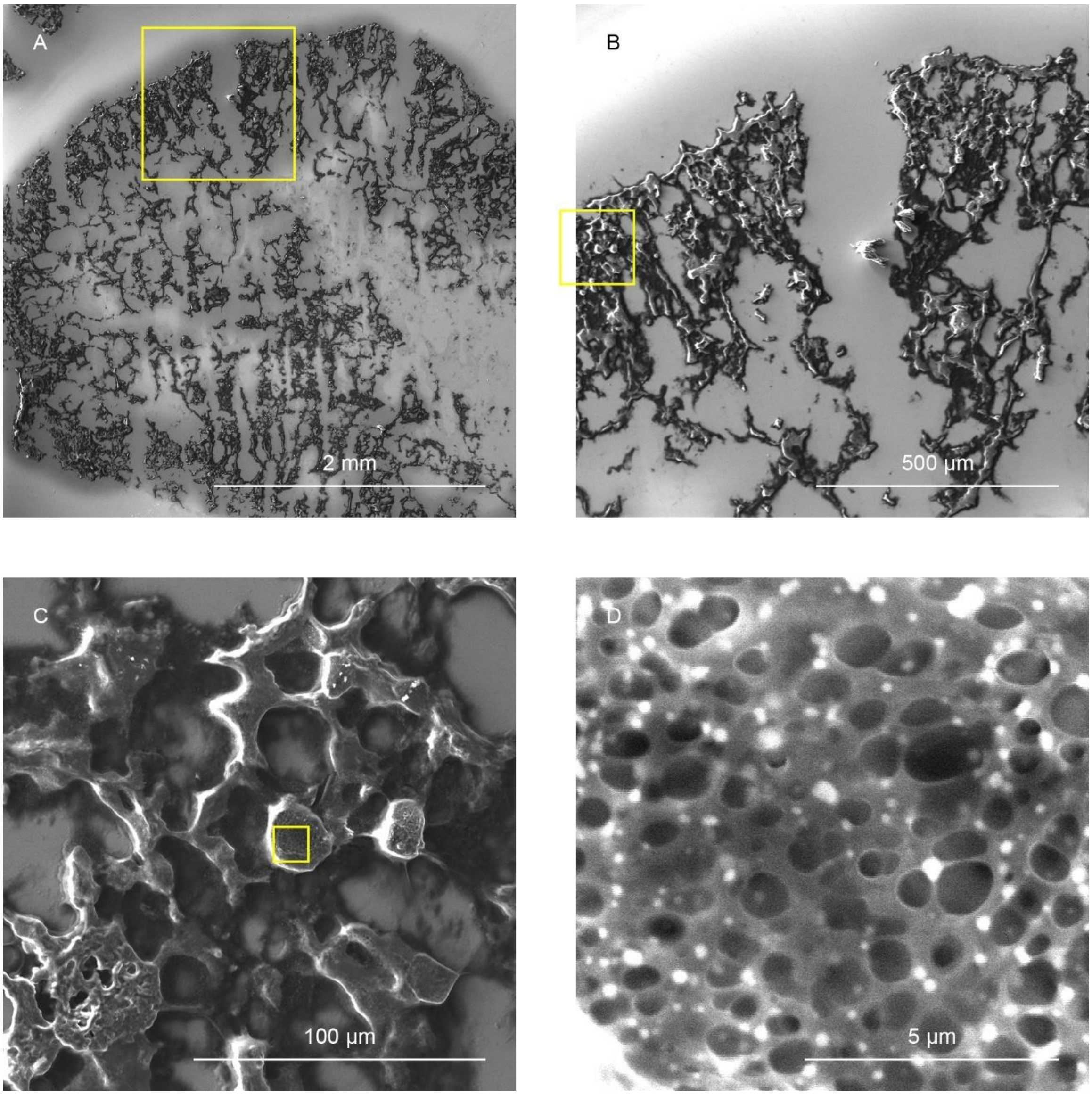

Figure S3. SEM microscopy images of the treated biofilm section. Images are from the biofilm analyzed with SIMS and are shown at different magnifications.

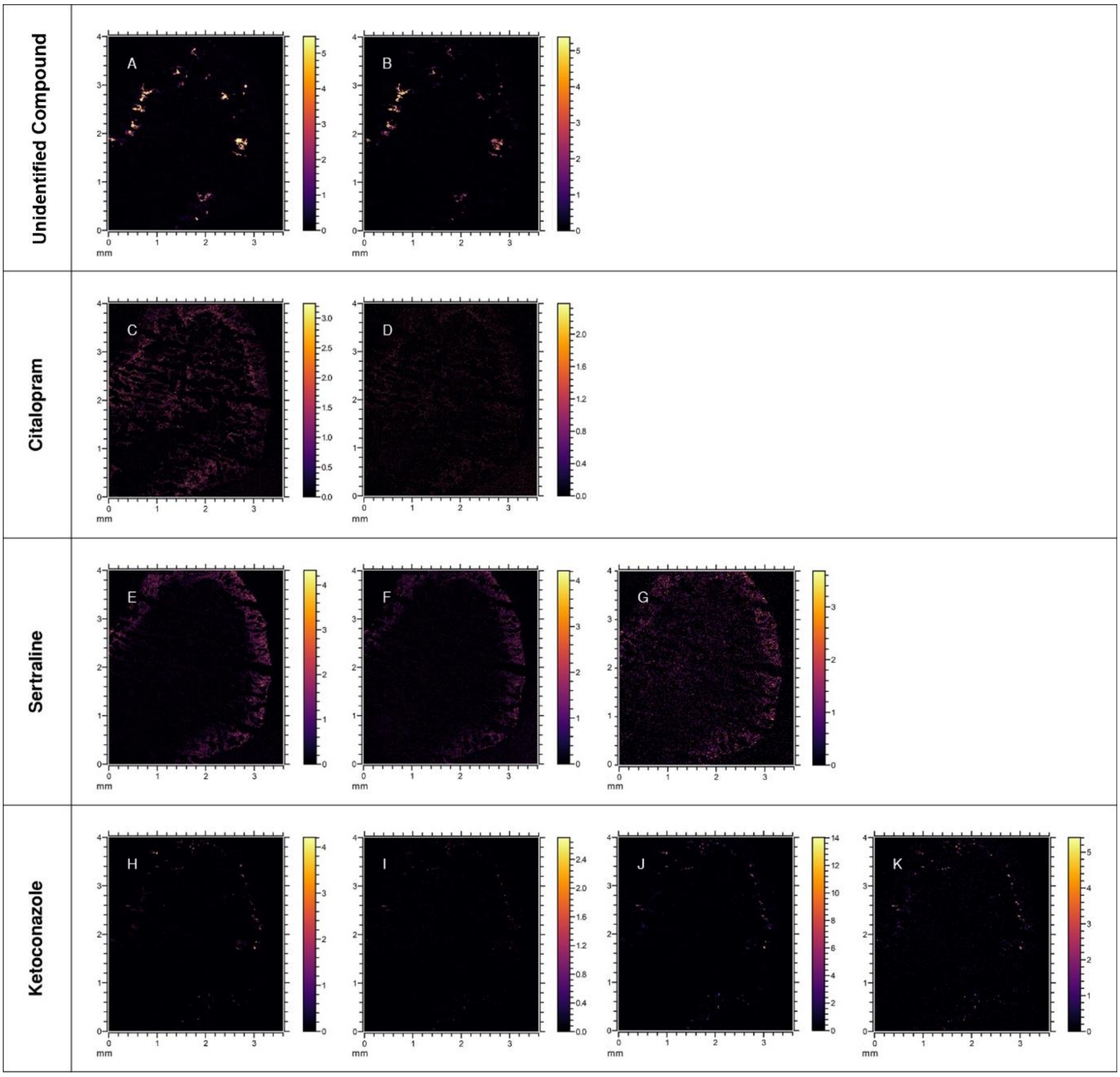

Figure S4. Distribution of identified peaks in the treated biofilm. Ion images of peaks *m/z* 243.0 (A) and 271.0 (B) with total ion counts of $5.1 \cdot 10^4$ and $3.0 \cdot 10^4$ respectively. Ion images of citalopram at peaks *m/z* 325.1 (C) and 326.1 (D) with total ion counts of $6.3 \cdot 10^4$ and $2.4 \cdot 10^4$ respectively. Ion images of sertraline at peaks *m/z* 275.0 (E), 277.0 (F), and 306.0 (G) with total ion counts of $6.4 \cdot 10^4$, $5.6 \cdot 10^4$, and $2.0 \cdot 10^4$ respectively. Ion images of ketoconazole at peak *m/z* 495.1 (H), and its transformation products at peaks 565.1 (I), 523.1 (J), and 537.1 (K) with total ion counts of $7.9 \cdot 10^3$, $3.5 \cdot 10^3$, $8.2 \cdot 10^3$, and $4.8 \cdot 10^3$ respectively. The color scale on the right indicates the relative SIMS signal intensity from high (white/yellow) to low (black/purple).

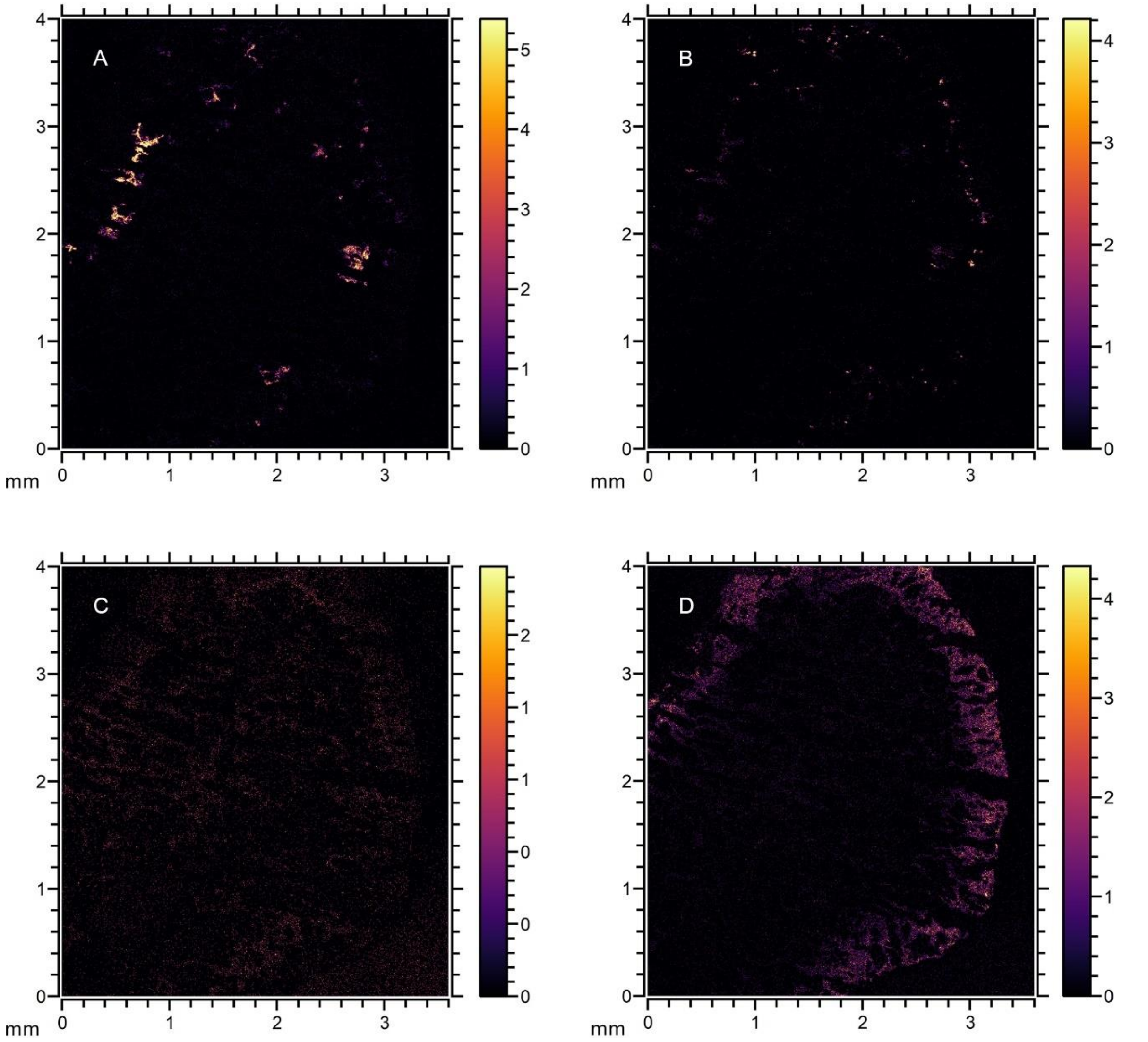

Figure S5. Images of representative *m/z* peaks obtained from the treated biofilm corresponding to *m/z* 271.0 (A), ketoconazole (B) at *m/z* 495.1, citalopram (C) at *m/z* 325.1, and sertraline (D) at *m/z* 275.0. The total ion counts for the selected *m/z* were $3.0 \cdot 10^4$, $6.3 \cdot 10^4$, $6.3 \cdot 10^4$, and $7.8 \cdot 10^3$, respectively. The color scale on the right indicates the relative SIMS signal intensity from high (white/yellow) to low (black/purple).

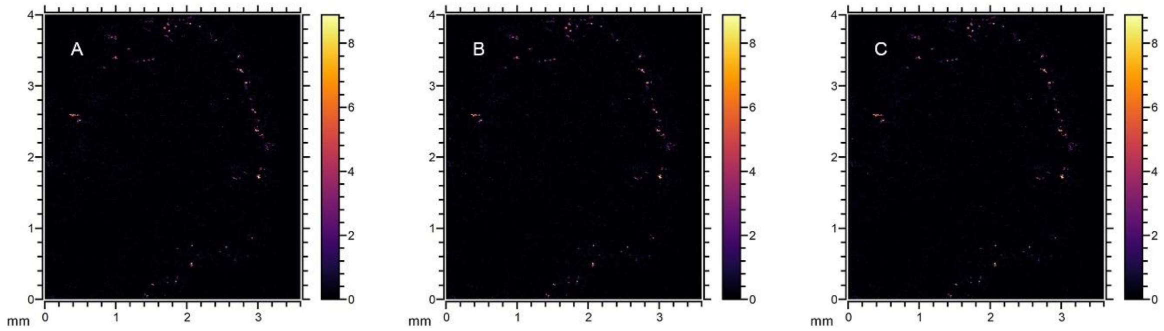

Figure S6. Distribution of unidentified peaks *m/z* 509.1 (A), 551.1 (B), and 579.1 (C) in the treated biofilm. Their distribution matches the peaks corresponding to ketoconazole and its TPs, suggesting that those might be fragments of ketoconazole metabolites. The color scale on the right indicates the relative SIMS signal intensity from high (white/yellow) to low (black/purple). The total ion counts for the selected *m/z* were $4.9 \cdot 10^3$, $4.5 \cdot 10^3$, and $1.3 \cdot 10^3$, respectively.

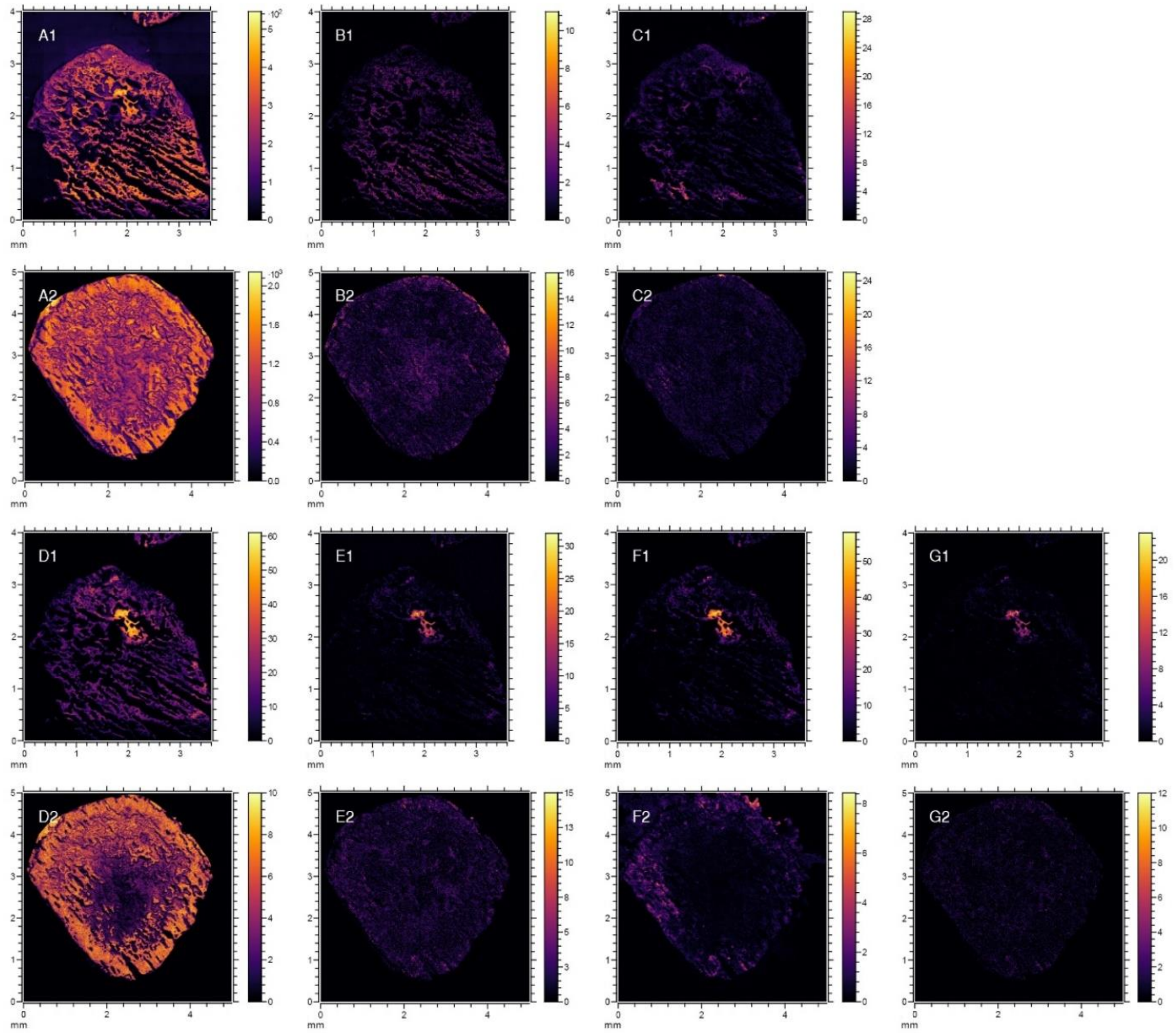

Figure S7. Ion images of the two control biofilms (1 and 2) depicting the total ion image (A), the distribution of adenine at *m/z* 136.0 (B), and lysine at *m/z* 147.1 (C), and the localization of phosphocholine headgroup at *m/z* 86.1 (D), 166.0 (E), 184.0 (F) and 224.0 (G). The total ion counts for *m/z* 136.0, 147.1, 86.1, 166.0, 184.0, and 224.0 in control 1, were $6.2 \cdot 10^4$, $1.3 \cdot 10^5$, $5.7 \cdot 10^5$, $9.9 \cdot 10^4$, $1.9 \cdot 10^5$, and $3.2 \cdot 10^4$, respectively. The total ion counts for *m/z* 136.0, 147.1, 86.1, 166.0, 184.0, and 224.0 in control 1, were $2.0 \cdot 10^5$, $2.1 \cdot 10^5$, $2.0 \cdot 10^6$, $1.5 \cdot 10^5$, $3.5 \cdot 10^5$, and $6.4 \cdot 10^4$, respectively.

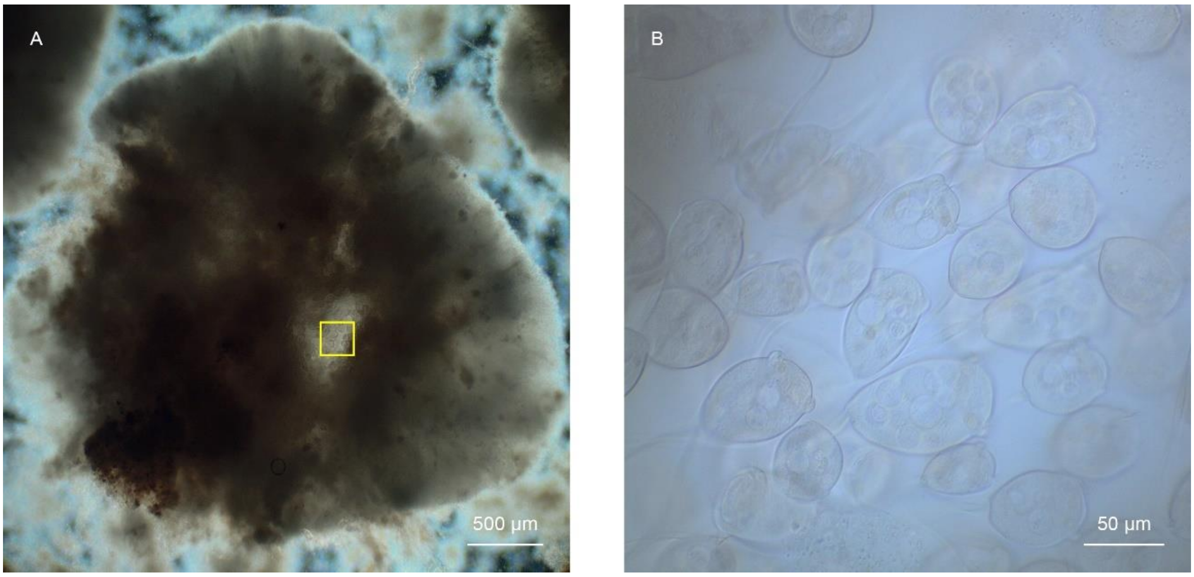

Figure S8. Light microscopy images of the cultivated aerobic granules with visible protozoa on the surface and within the matrix of the biofilm. The image on the left (A) was taken at 2x magnification (scale bar 500 µm). The image on the right (B) was taken at 20x magnification (scale bar 50 µm).

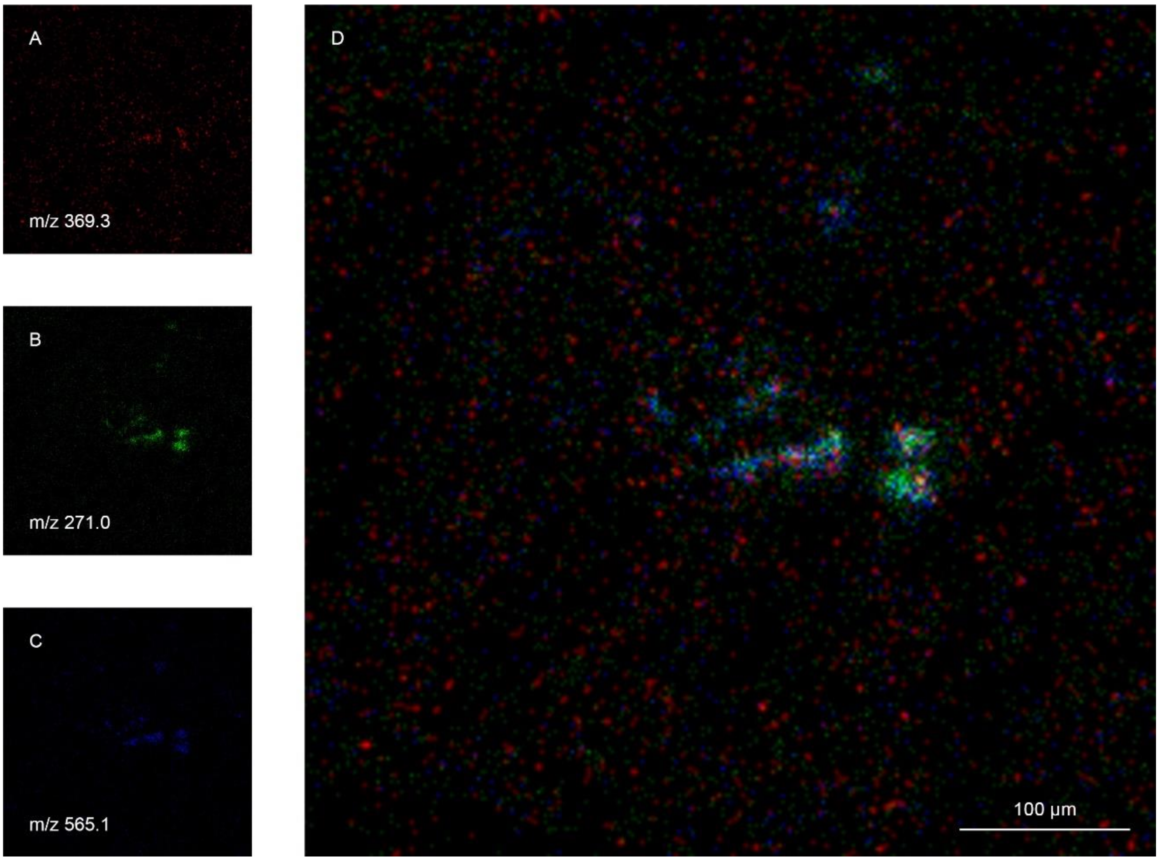

Figure S9. Ion images of peaks *m/z* 369.3 corresponding to cholesterol (A), *m/z* 271.0 (B), and *m/z* 565.1 representing ketoconazole (C). Overlay of the three peaks *m/z* 369.3, 271.0, and 565.1 (D).

SI References

(1) Layer, M.; Adler, A.; Reynaert, E.; Hernandez, A.; Pagni, M.; Morgenroth, E.; Holliger, C.; Derlon, N. Organic Substrate Diffusibility Governs Microbial Community Composition, Nutrient Removal Performance and Kinetics of Granulation of Aerobic Granular Sludge. *Water Res. X* **2019**, *4*, 100033. https://doi.org/10.1016/J.WROA.2019.100033.

(2) Park, J.; Yamashita, N.; Park, C.; Shimono, T.; Takeuchi, D. M.; Tanaka, H. Removal Characteristics of Pharmaceuticals and Personal Care Products: Comparison between Membrane Bioreactor and Various Biological Treatment Processes. *Chemosphere* **2017**, *179*, 347–358. https://doi.org/10.1016/J.CHEMOSPHERE.2017.03.135.

(3) Stevens-Garmon, J.; Drewes, J. E.; Khan, S. J.; McDonald, J. A.; Dickenson, E. R. V. Sorption of Emerging Trace Organic Compounds onto Wastewater Sludge Solids. *Water Res.* **2011**, *45* (11), 3417–3426. https://doi.org/10.1016/J.WATRES.2011.03.056.

(4) Golovko, O.; Örn, S.; Sörengård, M.; Frieberg, K.; Nassazzi, W.; Lai, F. Y.; Ahrens, L. Occurrence and Removal of Chemicals of Emerging Concern in Wastewater Treatment Plants and Their Impact on Receiving Water Systems. *Sci. Total Environ.* **2021**, *754*, 142122. https://doi.org/10.1016/j.scitotenv.2020.142122.

(5) Ternes, T. A.; Herrmann, N.; Bonerz, M.; Knacker, T.; Siegrist, H.; Joss, A. A Rapid Method to Measure the Solid–Water Distribution Coefficient (Kd) for Pharmaceuticals and Musk Fragrances in Sewage Sludge. *Water Res.* **2004**, *38* (19), 4075–4084. https://doi.org/10.1016/J.WATRES.2004.07.015.

(6) Wick, A.; Fink, G.; Joss, A.; Siegrist, H.; Ternes, T. A. Fate of Beta Blockers and Psycho-Active Drugs in Conventional Wastewater Treatment. *Water Res.* **2009**, *43* (4), 1060–1074. https://doi.org/10.1016/J.WATRES.2008.11.031.

(7) Radjenović, J.; Petrović, M.; Barceló, D. Fate and Distribution of Pharmaceuticals in Wastewater and Sewage Sludge of the Conventional Activated Sludge (CAS) and Advanced Membrane Bioreactor (MBR) Treatment. *Water Res.* **2009**, *43* (3), 831–841. https://doi.org/10.1016/j.watres.2008.11.043.

(8) Fernandez-Fontaina, E.; Pinho, I.; Carballa, M.; Omil, F.; Lema, J. M. Biodegradation Kinetic Constants and Sorption Coefficients of Micropollutants in Membrane Bioreactors. *Biodegradation* **2013**, *24* (2), 165–177. https://doi.org/10.1007/S10532-012-9568-3/TABLES/5.

(9) Polesel, F.; Lehnberg, K.; Dott, W.; Trapp, S.; Thomas, K. V.; Plósz, B. G. Factors Influencing Sorption of Ciprofloxacin onto Activated Sludge: Experimental Assessment and Modelling Implications. *Chemosphere* **2015**, *119*, 105–111. https://doi.org/10.1016/j.chemosphere.2014.05.048.

(10) Hörsing, M.; Ledin, A.; Grabic, R.; Fick, J.; Tysklind, M.; Jansen, J. la C.; Andersen, H. R. Determination of Sorption of Seventy-Five Pharmaceuticals in Sewage Sludge. *Water Res.* **2011**, *45* (15), 4470–4482. https://doi.org/10.1016/j.watres.2011.05.033.

(11) Fick, J.; Lindberg, R. H.; Kaj, L.; Brorström-Lundén, E. Results from the Swedish National Screening Programme 2010 Subreport 3. Pharmaceuticals. **2011**.

(12) Östman, M.; Lindberg, R. H.; Fick, J.; Björn, E.; Tysklind, M. Screening of Biocides, Metals and Antibiotics in Swedish Sewage Sludge and Wastewater. *Water Res.* **2017**, *115*, 318–328. https://doi.org/10.1016/J.WATRES.2017.03.011.

(13) Mohammadi, A. S.; Fletcher, J. S.; Malmberg, P.; Ewing, A. G. Gold and Silver Nanoparticle-Assisted Laser Desorption Ionization Mass Spectrometry Compatible with Secondary Ion Mass Spectrometry for Lipid Analysis. *Surf. Interface Anal.* **2014**, *46* (S1), 379–382. https://doi.org/10.1002/SIA.5609.

(14) Passarelli, M. K.; Winograd, N. Lipid Imaging with Time-of-Flight Secondary Ion Mass Spectrometry (ToF-SIMS). *Biochim. Biophys. Acta - Mol. Cell Biol. Lipids* **2011**, *1811* (11), 976–990. https://doi.org/10.1016/J.BBALIP.2011.05.007.

(15) Zhang, J.; Brown, J.; Scurr, D. J.; Bullen, A.; Maclellan-Gibson, K.; Williams, P.; Alexander, M. R.; Hardie, K. R.; Gilmore, I. S.; Rakowska, P. D. Cryo-OrbiSIMS for 3D Molecular Imaging of a Bacterial Biofilm in Its Native State. *Anal. Chem.* **2020**, *92* (13), 9008–9015. https://doi.org/10.1021/ACS.ANALCHEM.0C01125/SUPPL_FILE/AC0C01125_SI_004.AVI.

(16) Lanni, E. J.; Masyuko, R. N.; Driscoll, C. M.; Dunham, S. J. B.; Shrout, J. D.; Bohn, P. W.; Sweedler, J. V. Correlated Imaging with C60-SIMS and Confocal Raman Microscopy: Visualization of Cell-Scale Molecular Distributions in Bacterial Biofilms. *Anal. Chem.* **2014**, *86* (21), 10885–10891. https://doi.org/10.1021/AC5030914/SUPPL_FILE/AC5030914_SI_001.PDF.
